# Self-Supporting Ion Gels for Electrochemiluminescent Sticker-Type Optoelectronic Devices

**DOI:** 10.1038/srep29805

**Published:** 2016-07-15

**Authors:** Kihyon Hong, Yeong Kwan Kwon, Jungho Ryu, Joo Yul Lee, Se Hyun Kim, Keun Hyung Lee

**Affiliations:** 1 Surface Technology Division, Korea Institute of Materials Science (KIMS), Changwon 641-831, Republic of Korea; 2 Department of Chemical Engineering, Inha University, Incheon 402-751, Republic of Korea; 3 Functional Ceramic Group, Korea Institute of Materials Science (KIMS), Changwon 641-831, Republic of Korea; 4 Department of Nano, Medical and Polymer Materials, Yeungnam University, Gyeongsan 712-749, Republic of Korea

## Abstract

Nowadays, there has been an increasing demand to develop low-cost, disposable or reusable display devices to meet and maximize short-term user convenience. However, the disposable device has unfortunately not materialized yet due to the light-emitting materials and fabrication process issues. Here, we report sticker-type electrochemiluminescent (ECL) device using self-supporting, light-emitting gel electrolytes. The self-supporting ion gels were formulated by mixing a network-forming polymer, ionic liquid, and metal complex luminophore. The resulting ion gels exhibit excellent mechanical strength to form free-standing rubbery light-emitting electrolyte films, which enables the fabrication of sticker-type display by simple transfer and lamination processes on various substrates. The sticker-type ECL devices can be operated under an AC bias and exhibit a low operating voltage of 4 V (peak-to-peak voltage) with a maximum luminance of 90 cd/m^2^. It is notable that the result is the first work to realize sticker displays based on electrochemical light emitting devices and can open up new possibilities for flexible or disposal display.

Electrochemiluminescent (ECL) cells are light emitting devices that emit light through the radiative relaxation of excited luminophores that have been generated by electrochemical charge transfer reactions between reduced and oxidized species^1^,^2^,^3^,^4^,^5^. ECL devices have recently become a significant target of interest by researchers since these represent a possible alternative to organic light emitting diodes (OLEDs). In contrast to OLEDs – which consist of multiple stacks of organic layers, such as charge transport layers, charge injection layers, and emissive layers – ECL devices only need a single emissive layer sandwiched between two electrodes to support the entire luminescent process for light emission, including charge injection, transport, and electron-hole recombination^5^,^6^, and this simple device architecture (electrode/emissive layer/electrode) allows cost-effective device fabrication. In addition, the solution-based active layer deposition can eliminate complex and expensive vacuum processes with no restriction on the electrode materials. As a result, extensive researches have been conducted to determine the optimum emissive materials, device structure, and fabrication processes to obtain high performance ECL devices^7^,^8^,^9^.

Conventional ECL devices are composed of ionic transition metal complexes (ITMC) or conjugated polymeric (CP) semiconductors blended with supporting solid electrolytes based on Li salts and poly(ethylene oxide) host polymers^10^,^11^,^12^. Since most electrolytes used in previous works exhibit a low ionic conductivity, the resulting ECL devices suffer from a long response time that is larger than a few seconds^4^,^13^. To address this issue, a frozen junction concept was applied by utilizing permanently established ionic profiles at the device operation stage, particularly for CP-based light emitting devices^14^. For ITMC-based devices, ECL cells based on highly conductive electrolytes, including ionic liquid and ion gels, have been demonstrated^15^,^16^,^17^,^18^. ECL devices using ionic liquids or gel electrolytes have exhibited a sub-ms response time, suggesting that ion gel-based ECL devices can be strong candidates for use in high-speed electrochemical light emitting devices^19^,^20^,^21^.

A significant amount of research has been devoted toward developing the appropriate device fabrication processes to realize electronic devices with a large production area at a low cost. Specifically for ECL devices, various processing techniques have been utilized, including solution casting, spin coating, spray coating, inkjet printing, and slot-die coating^7^,^22^,^23^,^24^,^25^,^26^. For example, large area (ca. 14 × 14 cm^2^) ECL cells with a uniform light emission at a luminance of 200 cd/m^2^ were fabricated on flexible paper substrates by using spray sintering^23^. Inkjet printing was also employed to pattern lattice devices that displayed well-defined, high-contrast static letters with a pixel density of 170 PPI^26^. For continuous fabrication, a slot-die roll coating was used to deposit whole device layers with a light emitting polymer composite and two electrodes at a process rate of 0.6 m/min^24^. In conjunction with such improvements, there has been an increasing demand to develop low-cost disposable or reusable electronic devices to meet and maximize short-term user convenience. As a result, various types of disposable transistors, biosensors, and acoustic wave resonators have been developed^27^,^28^,^29^. However, disposable ECL devices or throwaway displays have not been previously reported.

In this paper, we demonstrate sticker-type ECL devices with self-supporting light-emitting electrolytes that can be simply transferred and laminated several times on various substrates. The self-supporting ion gels were formulated by mixing a network-forming polymer – poly(vinylidene fluoride-*co*-hexafluoropropylene) (P(VDF-HFP)) – and ionic liquid – 1-ethyl-3-methylimidazolium bis(trifluoromethylsulfonyl)imide ([EMIM][TFSI]). A transition metal complex luminophore, Ru(bpy)_3_Cl_2_ was incorporated into the ion gel to confer the light-emitting characteristic^3^,^21^,^30^. The resulting ion gels exhibit excellent mechanical strength to form free-standing rubbery light-emitting electrolyte films, which enables the fabrication of sticker-type ECL devices on flexible substrates. More importantly, ECL gels can be detached and re-adhered to the substrates multiple times, ca. 5 times, similar to a reusable “Post-it” note. Light was successfully generated using various electrodes, including indium tin oxide (ITO), Ag nanowire (NW), Au metal mesh grid, and stainless steel. The sticker-type ECL devices can be operated under an AC bias and exhibit a low operating voltage of 4 V (peak-to-peak voltage) with a maximum luminance of 90 cd/m^2^. To the best of our knowledge, this is the first work to realize sticker displays based on electrochemical light emitting devices. Thus, we believe that sticker-type ECL devices can be applied toward various uses, including wearable devices and ubiquitous stick-and-display systems on various substrates where direct fabrication is not possible.

## Results

### Fabrication process of sticker ECL device

**Figure 1:**
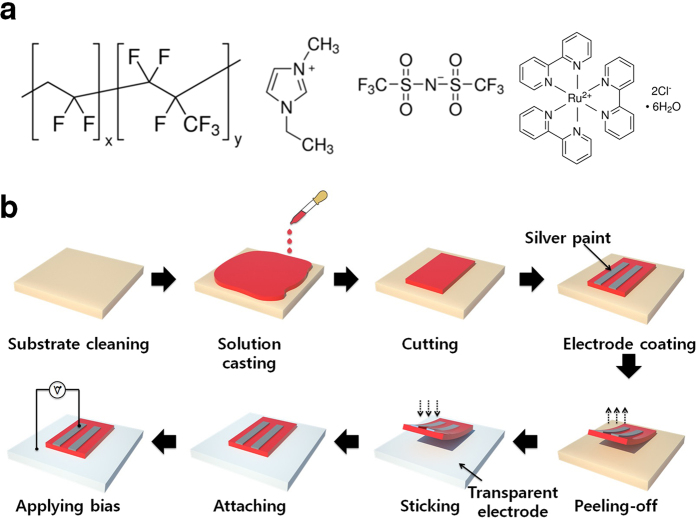
(**a**) Molecular structures of the ion gel film components, P(VDF-HFP), [EMIM][TFSI], and Ru(bpy)_3_Cl_2_. (**b**) Schematic illustration to fabricate a sticker type ECL device using a light-emitting ion gel. The ion gel solution is cast on a substrate such as glass, Si wafer, and plastic film. As a top electrode, the Ag layer is coated via simple brush painting. The dried and free-standing ion gel film can be transferred onto target substrates with various transparent electrode materials, including ITO, Ag NW, and Au mesh grid.

A sticker ECL device can be fabricated by simply peeling-off and attaching the self-supporting light-emitting ion gel film to the desired surface (Fig. 1, Supporting Information Video 1). The fabrication process begins by solution casting the ion gel ink onto a substrate. The gel ink was prepared by co-dissolving P(VDF-HFP), [EMIM][TFSI], and Ru(bpy)_3_Cl_2_ in acetone (Fig. 1a). The resulting ion gel film was cast and further dried in ambient conditions for 24 h to remove the residual solvent. The thickness of the resulting ion gels was measured to be ~400 μm. The resulting ion gel samples can be cut with a blade and peeled-off using tweezers because of the outstanding mechanical integrity of the ion gel (Supporting Information, Figure S1). A Young’s modulus value of 1.1 ± 0.3 MPa was measured from ECL ion gels which is around 2 orders of magnitude higher than the typical modulus of the ion gels with ABA block copolymer at the same polymer concentrations^31^,^32^,^33^. Note that the gel modulus remains almost the same after application of external voltages, *i*.*e*. ECL measurements. To form the top electrode of an ECL device, an Ag layer was coated by simple brush painting. To fabricate the ECL devices, the peeled-off ion gels were transferred onto transparent receiving electrodes. Note that the sticker-type ion gel can be attached onto various target substrates, including glass, Si wafer, metal foil, and flexible polyethylene terephthalate (PET) film (Supporting Information Figure S2). The luminance (*L*)*-*current (*I*)*-*voltage (*V*) characteristics of the devices were then measured in ambient air to evaluate the electroluminescent property of the sticker-type ECL device.

### Characterization of free-standing ion gels

**Figure 2:**
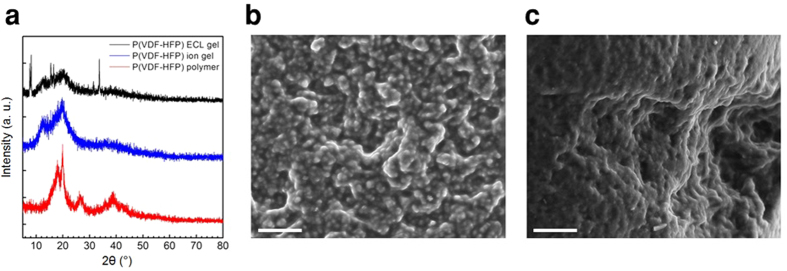
(**a**) X-ray diffraction spectra of the light emitting P(VDF-HFP) ECL gel (top), normal P(VDF-HFP) gel (middle), and P(VDF-HFP) polymer (bottom). Strong diffraction peaks and the corresponding d-spacing values for the PVDF crystals are listed in Table S1 of the Supporting Information. For clarity, the XRD spectra are arbitrarily shifted vertically. SEM images collected from a PVDF-HFP ion gel (**b**) and for a light emitting ECL gel (**c**). Scale bars are 10 μm.

The physical origins of the gel formation are investigated using X-ray diffraction (XRD) and scanning electron microscope (SEM) analyses on a luminophore containing ion gel, a normal ion gel, and a reference film of pure P(VDF-HFP) polymer (Fig. 2). The main diffraction peaks and the corresponding crystallographic planes are listed in Table S1 of the Supporting Information. The diffraction patterns in Fig. 2a suggest that the crystallinity of the gel samples decreases by blending the ionic liquid with the host polymer. This observation is corroborated with a reduction in the melting and crystallization peaks observed in the differential scanning calorimetry (DSC) thermogram upon the addition of [EMIM][TFSI] (Supporting Information, Figure S3). The XRD patterns that were measured reveal that the VDF segments in the copolymer tend to generate different crystal polymorphs under different conditions. The solvent, solvent-evaporation rate, mechanical deformation, and electric field are well-known to affect the type of predominant polymorph in PVDF crystals^34^,^35^,^36^,^37^,^38^.

Strong peaks for the pure polymer at 2θ = 17.9, 19.9, 26.5, and 38.7° correspond to the (100)/(020), (110), (021) and (002)/(131) planes of the α phase PVDF crystals, which is the most stable phase at ambient conditions^37^. Upon the addition of the ionic liquid [EMIM][TFSI], the diffraction peaks become more broad, and the peaks at around 26.5 and 38.7° are greatly suppressed for both normal and light emitting ion gels. The peak at ~20° is typical of the (101) plane of the γ polymorph and can be observed in both ion gels. In addition, a new peak at a low diffraction angle of ~13° corresponds to the d-spacing value of ~7 Å^37^. Considering that ferroelectric β and γ phases can be obtained by blending [EMIM] cation-containing ionic liquids and that the amount of γ phase increases when the size of anion increases from [NO_3_] to [CF_3_SO_3_] and to [C_2_H_5_SO_4_], the peak at 13° might be assigned to the (011) plane of the orthorhombic γ phase of the PVDF crystals^38^. Note that the van der Waals volume of the TFSI calculated by the *ab initio* molecular orbital simulation is 147 Å^3^, and this value is expected to be larger than that for the anions listed above^39^. Therefore, the charged cations and anions in ionic liquid might induce permanent dipoles in the crystals, which results in a ferroelectric γ polymorph rather than the non-polar and paraelectric α phase PVDF.

The SEM images in Fig. 2b,c clearly display phase-separated PVDF crystals of both ion gels with a size of a few μm, and these crystalline domains act as physical crosslinking cores for the 3D polymer network in the solid ion gels. The gel forms by connecting those micellar cores with polymer chains dissolved in the ionic liquid. Note that for the concentrated light emitting gels, phase-separated angular crystals – which we expect to be Ru(bpy)_3_Cl_2_ – were also observed. Such luminophore crystals account for the sharp peaks in the XRD of the ECL gels (see Fig. 2a and Supporting Information Figures S4a and S4b). The gel morphology and the device performance can be further optimized since these crystals can be re-dissolved into the gel matrix upon thermal annealing of the sample at elevated temperatures, ca. 130 °C, as shown in the Supporting Information Figure S4c. After application of external voltages, *i*.*e*. ECL measurements, the gels look qualitatively the same and no significant changes were observed in the gel morphology (Supporting Information, Figure S5).

The thermal stability of the ion gels and the host polymer was characterized via thermogravimetric analysis (TGA) under a nitrogen atmosphere with a heating rate of 10 °C/min (see Supporting Information Figure S6), and the light emitting gel is determined to be thermally stable above 250 °C. Based on the weight fraction of the luminophore and the thermal stability of the normal ion gels, the initial weight loss at ~250 °C is a result of the decomposition of the Ru transition metal complex, and the ion gel without the luminophore is thermally stable at up to ~300 °C.

### Operating mechanism for the ion gel based ECL device

**Figure 3:**
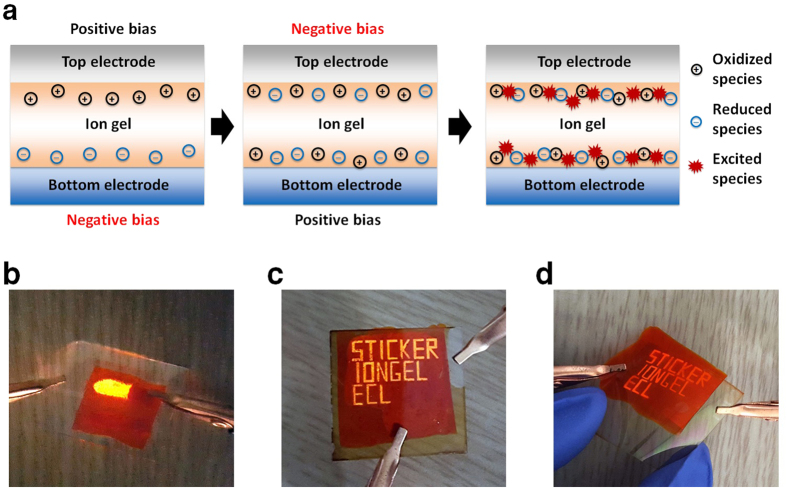
(**a**) Schematic diagram of the chemical species and electrochemical processes in an ECL device under an AC bias. The photographs of the ON state for (**b**) a single sticker ECL pixel, (**c**) patterned letters of the “STICKER IONGEL ECL” on glass/ITO and (**d**) static letters on a bendable PET/ITO substrate. The applied voltage and frequency were V_PP_ = 3.6 V and 1 Hz, respectively.

The operating mechanism for the ion gel based ECL device can be explained to be a result of the electrochemical redox reaction between the reduced and oxidized luminophores (Fig. 3a)^40^,^41^. Typical ECL devices can operate using both DC and AC. When an external bias is applied between the two electrodes, the luminophores are oxidized and *p*-type carriers are introduced at anode, whereas the luminophore adjacent to the cathode is reduced and *n*-type carriers are injected into the active layer. For the redox species to react with each other, both *p*-/*n*-type carriers must diffuse from each electrode to the center of the light-emitting electrolyte. The slow diffusion of the redox species and the low ionic conductivity of polymeric solid electrolytes result in DC-driven ECL devices typically showing a slow response time (~a few seconds) that is not favorable for practical applications. When an AC bias is applied to the ECL cells, the reduced and oxidized species, Ru(bpy)_3_^1+^ and Ru(bpy)_3_^3+^, respectively, can be continuously generated and recombined near the electrode/ion gel interfaces due to the bias polarity that switches reversibly^2^. As a result, an excited state Ru(bpy)_3_^2+*^ is efficiently generated near the interfaces of both electrodes, and the device emits light with a relatively short operating time. In this regard, sticker ECL cells on a glass or PET substrate were tested under an AC bias, and Fig. 3b–d show the patterned lattice devices that displayed three rows of well-defined, high-contrast static letters: “STICKER IONGEL ECL”.

### Electroluminescent properties of sticker-type optoelectronic device

The optimum ECL device performance is obtained by investigating the luminance-voltage (square wave peak-to-peak voltage, V_PP_) characteristic of the devices with different Ru(bpy)_3_Cl_2_ concentrations, fabricated on a glass/ITO substrate. As shown in Figure S7a, most devices exhibit a low turn-on voltage of about V_PP_ = 2.0 V and a maximum luminance at 3.6~4.0 V. At higher voltage above V_PP_ = 4.0 V, the device exhibited reduced luminance. This phenomenon is related to the electrochemical window (EW) of ionic liquid in the ion gel. Typically, ionic liquids have EW in the range of 3–6 V. For [EMIM][TFSI], the EW value is about 4.19 V^42^. Thus, the ECL devices need to be operated in the EW of the ionic liquid to eliminate the possible degradation of the component ions. It is noteworthy the device stability can be improved by designing the component ions in the electrolyte^43^. The luminance improved as the concentration of Ru(bpy)_3_Cl_2_ increased, and for the 10 wt%-sample, the device showed a maximum luminance of 90 cd/m^2^ at V_pp_ = 3.6 V (*f* = 1 Hz). When the concentration further increased to 15 wt%, the luminance decreased to 70 cd/m^2^. This phenomenon might have originated from the segregation of Ru(bpy)_3_Cl_2_ and quenching effect induced by highly concentrated Ru(bpy)_3_Cl_2_ molecules. In addition, we investigated the effect of AC bias frequency on the luminance of the ECL device (Supporting Information Figure S7c). When we applied V_pp_ = 3.6 V with *f* = 1 Hz, the device exhibited a luminance of about 90 cd/m^2^. However, the device showed a decrease in luminance at a high frequency, which is generally the case for ECL systems^21^. Such behavior can be explained by the limited ionic motion in the emitting gels. When an external low frequency bias is applied to the system, the Ru(bpy)_3_^+^ and Ru(bpy)_3_^3+^ species are fully generated, and the electron transfer forms Ru(bpy)_3_^2+*^ and results in a bright emission. At a high frequency, however, the reduced and oxidized species do not have sufficient time to be generated and stabilized, resulting in a low luminance. This situation can be improved by increasing the ionic conductivity of the gels and by employing thinner ion gel films. The peak position and shape of the electroluminescent spectra for the ECL devices were not changed when various bias frequencies were applied, showing a maximum intensity at λ = 630 nm (Inset in Figure S7c). The device current in Supporting Information (Figure S7d) shows sharp faradaic current flowing peaks with alternating external voltages. The static current of the device is in the range from 3 to 25 mA (active area: 75 mm^2^), meaning that the static power consumption of a sticker-type ECL device is less than 45 mW.

We evaluated the long-term stability of ECL devices (Supporting Information, Figure S8). The measurement was performed in ambient condition. The device loses its luminance feature rather quickly and such a short life time of ECL devices has been observed in ITMC-based devices. The poor stability of ECL device is probably originated from the Ru(bpy)_3_Cl_2_ which has poor humidity-oxidation stability because the other components, P(VDF-HFP) and ionic liquids are hydrophobic and electrochemically stable at V_PP_ < 3.0 V^44^. We think that this situation can be improved by replacing the chloride salt complex to more stable luminophore materials^45^.

**Figure 4:**
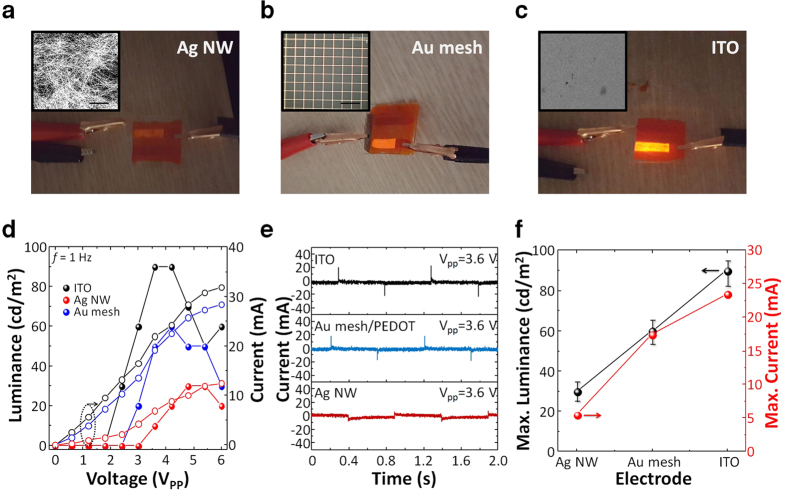
(**a–c**) Photographs of sticker ion gel ECL devices transferred onto various transparent electrode materials: Ag NW, Au mesh grid, and ITO. Inset: SEM or OM images for the surfaces of Ag NW (scale bar: 5 μm), Au mesh grid (scale bar: 400 μm), and ITO electrodes. (**d**) Voltage-luminance (closed symbols)-current (open symbols) (*f* = 1 Hz) and (**e**) current (V_pp_ = 3.6 V, *f* = 1 Hz) characteristics of ECL devices on various electrode materials. (**f**) Optical and electrical characteristics of ion gel ECL devices on Ag NW, Au mesh grid, and ITO electrodes (V_pp_ = 3.6 V, *f* = 1 Hz).

To confirm the universality and versatility of the peeling-off and sticking processes for the ECL device fabrication, various combinations of electrode materials were employed, with Ag NW, Au mesh grid, ITO, and stainless steel as target substrates, and the results are presented in Fig. 4. The active area of the devices was 75 mm^2^. The ECL devices using spin-coated Ag NW electrodes showed a relatively low maximum luminance of 30 cd/m^2^ with a low current level of ±6 mA (Fig. 4a,d–f). This is probably due to the low contact area, large surface roughness, and low corrosion resistance to the ionic liquid of the Ag electrode. The Ag NW has nm-scale diameter and μm-scale length. Under AC bias, strong voltage potential and electric field are applied to the Ag NW. Thus, the Ag NW which is electropositive metal can be oxidized easily (e.g. Ag → Ag^−^+e^−^) when it contact with an ion gel, leading to poor electroluminescent property of ECL device^46^. For the Au mesh grids electrodes (a square mesh with repeated period of 200 μm and width of 20 μm), a conductive polymer, poly(3,4-ethylenedioxythiophene):poly (styrenesulfonate) (PEDOT:PSS) was spin-coated on the Au mesh to cover the empty area and thereby lower the sheet resistance of the electrode. The device showed maximum luminance of 60 cd/m^2^ at a current level of ±18 mA. After the operation, the resistance of PEDOT:PSS film was slightly increased from 2.9 to 3.1 kohm, indicating the good oxidative stability of PEDOT:PSS during the device operation. The ECL devices on ITO exhibited the highest luminance value of 90 cd/m^2^ with a current level of ±25 mA due to the low sheet resistance (~10 ohm/sq), low surface roughness (<10 nm), and good electrochemical stability of the ITO film. The current and power efficiencies were 0.21 cd/A and 0.18 lm/W (at V_PP_ = 3.6 V and *f* = 1 Hz), respectively (Supporting Information, Figure S9). Finally, the sticker-type ECL devices were demonstrated on a stainless steel cup. The ion gel was transferred directly, and it adhered onto the surface of the electrically conductive stainless steel cup without any interlayer or sacrificial layer and a patterned PET/ITO electrode laminated on the top surface of ion gel (Supporting Information, Figure S10). The stainless steel cup/emitting gel/ITO devices exhibited luminance of 75 cd/m^2^ with a bright red light under an AC potential.

### Performance and repeatability of sticker ECL device

**Figure 5:**
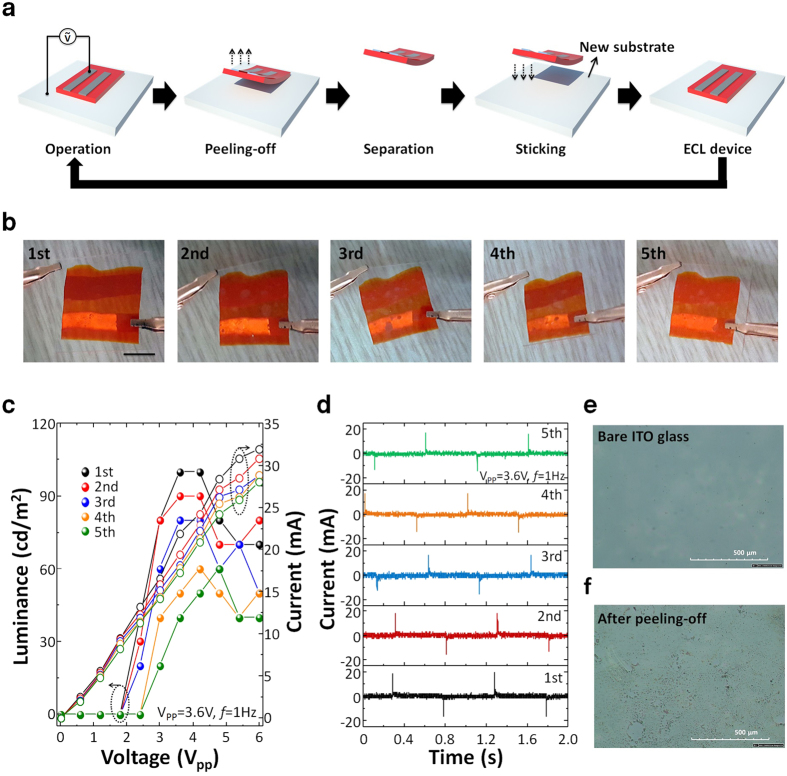
(**a**) Schematic of a repeatability test for sticker ECL devices. (**b**) Photographs taken in each repeatability test step (V_pp_ = 3.6 V, *f* = 10 Hz). Scale bar: 1 cm. Change in (**c**) voltage-luminance (closed symbols)-current (open symbols) curves and (**d**) current profile for five consecutive repeatability tests (V_PP_ = 3.6 V, *f* = 1 Hz). Optical microscope surface images of ITO glass (**e**) before and (**f**) after peeling-off the ion gel. Glass/ITO was used for the substrates.

To evaluate the repeatability of the sticker ECL device, five ITO substrates were tested with five successive peeling-off and sticking process (Fig. 5 and Supporting Information Figure S11 and Video 2). With this simple “Post-it” fabrication process, the device still responded well to a 1 Hz square wave input signal with V_pp_ = 3.6 V. The drop in the current level and luminance was observed, which is probably due to the ion gel residue teared out from the bulk, implying that pure ionic liquid or tiny ion gel pieces with poor connectivity can remain on the substrate after repeated attaching and peeling-off processes. The reliability and performance of the ECL devices could be improved by employing the appropriate structuring polymer networks, which can improve the mechanical integrity of the ion gel layer.

## Conclusion

In conclusion, we have successfully demonstrated the fabrication of sticker-type ECL devices using free-standing ion gels. The ion gels can be easily prepared by mixing matrix polymer (P(VDF-HFP)), ionic liquid ([EMIM][TFSI]), and a light emitting transition metal complex (Ru(bpy)_3_Cl_2_). The thickness of the resulting ion gels was measured to be ~400 μm and the amount of Ru(bpy)_3_Cl_2_ was calculated to be 2.7 mg/cm^2^. A simple solution casting, peeling-off and attaching strategy works very well for the sticker ECL devices, and the ion gel can be transferred onto glass and PET substrates with various electrode materials, including ITO, Ag NW, and an Au mesh grid. All ECL devices exhibited a low operating voltage of 4.0 V, and the ITO-ECL cell showed a maximum luminance of 90 cd/m^2^ at λ = 630 nm. The maximum static current of the device was of 25 mA, meaning that the static power consumption of the sticker-type ECL device was of less than 45 mW. Furthermore, the self-supporting ECL devices operated reasonably well after consecutive device fabrication by conducting a peeling off and re-attaching processes. We successfully showed the possibility of sticker-type display devices using ion gel electrolyte and the next step will thus be to extend the available target substrates to any kinds of unconventional surfaces by constructing conducting electrodes using spray coating. We believe that the strategy demonstrated in this paper can thus provide a new platform for reusable and disposable display devices.

## Methods

### Materials

Poly(vinylidene fluoride-*co*-hexafluoropropylene), P(VDF-HFP) with M_n_ = 130 000 g mol^−1^ and Ru(bpy)_3_Cl_2_ (purity: 99.7%) were purchased from Sigma-Aldrich. 1-Ethyl-3-methylimidazolium bis(trifluoromethylsulfonyl)imide, [EMIM][TFSI], was purchased from EMD Chemicals.

### Ion gel preparation

The free-standing ion gel was solution cast on a glass slide. The light emitting ion gel solution was prepared by codissolving P(VDF-HFP), [EMIM][TFSI], Ru(bpy)_3_Cl_2_ in acetone. The weight ratio between the polymer, ionic liquid, and the solvent was kept at 1:4:7. The concentration of Ru(bpy)_3_Cl_2_ was varied from 2 to 15 wt% to optimize the ECL device performance. The solution was stirred at room temperature for 2 hr. The preparation procedure for normal ion gels without Ru(bpy)_3_Cl_2_ was the same as that for light emitting ion gels.

### Ion gel characterization

The thermal characteristics of the light emitting ion gel, normal ion gel and P(VDF-HFP) polymer were analyzed via differential scanning calorimetry (DSC) and thermogravimetric analysis (TGA). The DSC curves were measured in the second heating and cooling scans by using a DSC 200 F3 (NETZSCH) in the temperature range from −70 to 200 °C at a rate of 10 °C/min. The TGA curves were obtained using a STA 409 PC (NETZSCH) in the temperature range from 25 to 600 °C at a heating rate of 10 °C/min under a nitrogen atmosphere. The morphology of the samples was characterized using a JEOL JCM-5000 Scanning Electron Microscope (SEM) and DMAX-2500 (Rigaku) X-ray diffractometer (XRD) in the angular range of 2θ = 5 to 80°. The DSC, TGA, XRD measurements were conducted in the Research Institute of Standards and Analysis at Inha University.

### Sticker-type ECL device fabrication and characterization

The solution cast ion gel film was dried at room temperature for 24 h to remove the residual solvent. Then, the dried ion gel was cut with a razor blade. The top electrode of ECL devices was formed with an Ag layer coated via simple brush painting. The ion gel sample is free-standing, can be peeled-off using tweezers and can then be transferred onto a new substrate with various transparent electrodes, including Ag NW, Au mesh grid, and ITO on glass or PET substrates. The Ag NW film was prepared using a simple spin coating method. The Au mesh grid could be fabricated using conventional photolithography, metal deposition, and lift-off processes. To reduce the sheet resistance and to increase the contact area, a conductive polymer, PEDOT:PSS, was spin-coated on the Au mesh grid. The luminance of the ECL device was measured using a Konica Minolta CA-210 device. The electrical properties of the ECL device were measured using Keithley 3390 arbitrary waveform generator and LeCroy WaveSurfer 44Xs-A oscilloscope with CP030A probe.

## Additional Information

**How to cite this article**: Hong, K. *et al*. Self-Supporting Ion Gels for Electrochemiluminescent Sticker-Type Optoelectronic Devices. *Sci. Rep.* **6**, 29805; doi: 10.1038/srep29805 (2016).

## Supplementary Material

Supplementary Information

Supplementary Video 2

Supplementary Video 1
