# Supplementary material for: Self-Supporting Ion Gels for Electrochemiluminescent Sticker-Type Optoelectronic Devices
Source: Sci Rep. 2016 Jul 15;6:29805. doi: 10.1038/srep29805 (PMC4945944; doi:10.1038/srep29805)
Supplement: Supplementary Information [file srep29805-s1.doc]

Supporting Information

Self-Supporting Ion Gels for Electrochemiluminescent Sticker-Type Optoelectronic Devices

Kihyon Hong*, Yeong Kwan Kwon, Jungho Ryu, Joo Yul Lee, Se Hyun Kim, and Keun Hyung Lee*


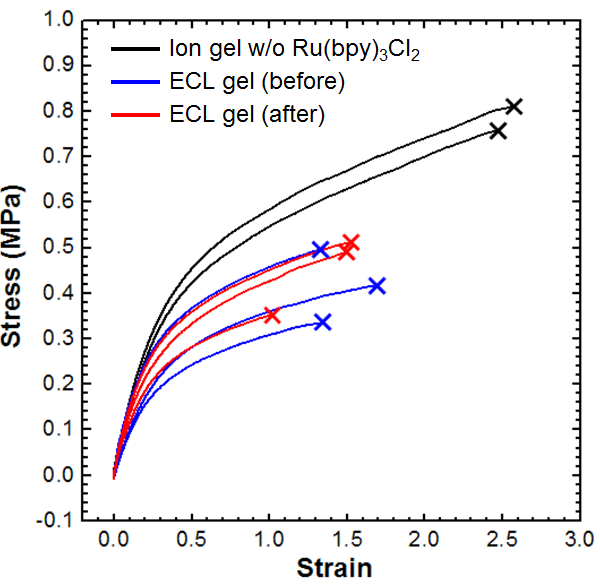


**Figure S1.** Stress versus stain curves for the normal ion gel without Ru(bpy)_3_Cl_2_ luminophore, and ECL gels before and after ECL measurements. Young’s Modulus values are 1.4 ± 0.1, 1.1 ± 0.3, and 1.2 ± 0.2 MPa for the normal gel, ECL gel before measurement and ECL gel after measurement, respectively. Strain at break values are 2.5 ± 0.1, 1.4 ± 0.2, and 1.3 ± 0.3 for the normal gel, ECL gel before measurement and ECL gel after measurement, respectively. Outstanding Young’s modulus values above 1 MPa enable the gels to be easily peeled-off and re-adhered on various substrates like a post-it sticker.


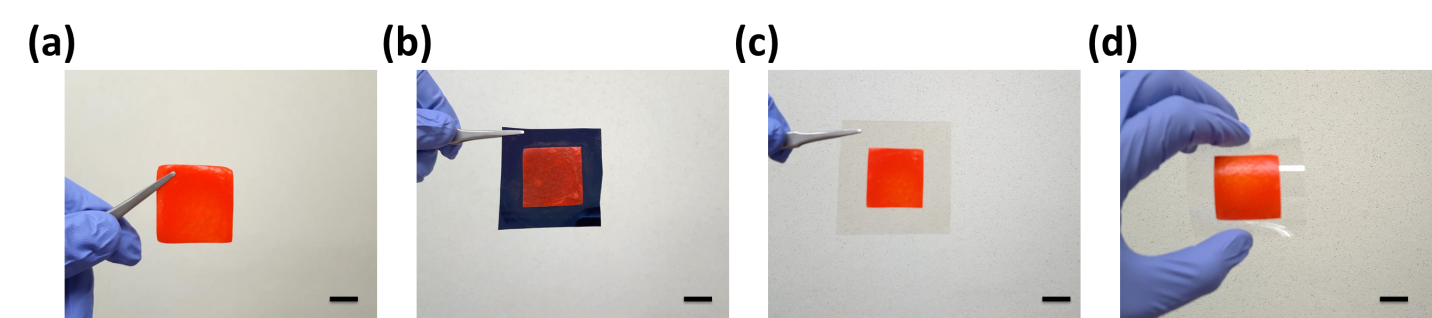


**Figure S2**. Photographs of (a) a free-standing ion gel film and the attached gel on (b) Si wafer, (c) glass, and (d) flexible PET.

| **Sample** | **2θ (°)** | **d-spacing (Å)** | **Plane** | **Phase** |
| --- | --- | --- | --- | --- |
| P(VDF-HFP) polymer | 17.9 | 4.94 | (1 0 0)  (0 2 0) | α |
|  | 19.9 | 4.46 | (1 1 0) |  |
|  | 26.5 | 3.36 | (0 2 1) |  |
|  | 38.7 | 2.32 | (0 0 2)  (1 3 1) |  |
| P(VDF-HFP) ion gel | 12.6 | 7.03 | (0 1 1) | γ |
|  | 19.8 | 4.47 | (1 0 1) |  |
|  | 38.6 | 2.33 | (0 4 1)  (1 3 2) |  |
| P(VDF-HFP) ECL gel | 12.7 | 6.97 | (0 1 1) | γ |
|  | 19.8 | 4.49 | (1 0 1) |  |
|  | 38.7 | 2.32 | (0 4 1)  (1 3 2) |  |

**Table S1**. X-ray diffraction peak positions (**2θ**), d-spacing (Å), and corresponding crystallographic planes for P(VDF-HFP) polymer, normal P(VDF-HFP) ion gel, and light emitting P(VDF-HFP) ECL gel. The crystalline polymorphs for each sample were labeled accordingly.


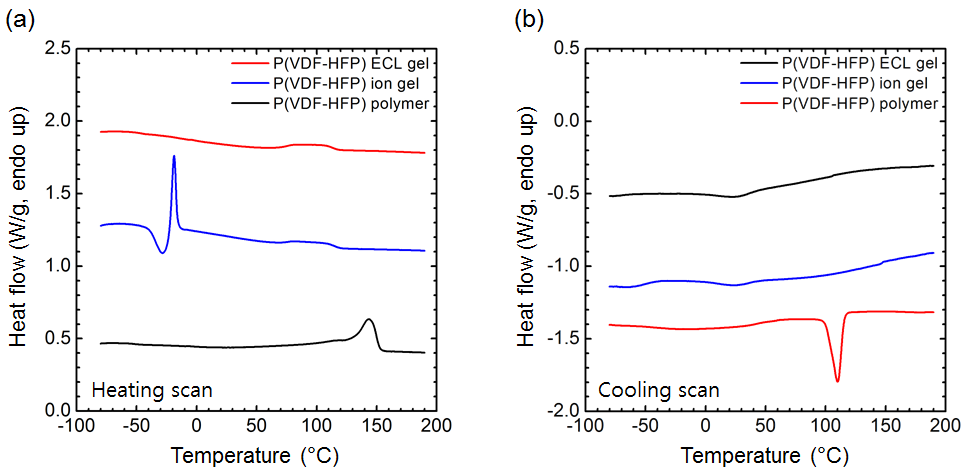


**Figure S3.** DSC thermogram of the light emitting P(VDF-HFP) ECL gel, P(VDF-HFP) ion gel and P(VDF-HFP) polymer. For clarity, the curves are arbitrary shifted vertically. DSC measurements were conducted from –80 °C to 200 °C at heating and cooling rates of 10 °C min^−1^. The curves were collected during the 2nd heating (a) and cooling scans (b) to remove any prior thermal history.


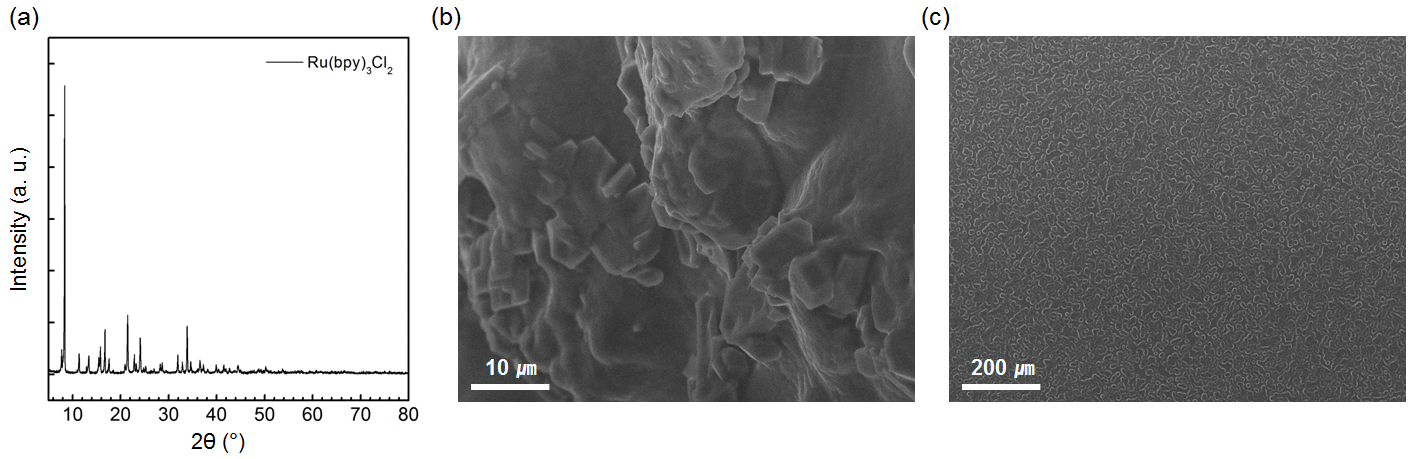


**Figure S4.** (a) X-ray diffraction spectra of the light emitting Ru(bpy)_3_Cl_2_ luminophore powder. SEM images collected from a concentrated P(VDF-HFP) ECL gel (b) and for an annealed ECL gel at 130 °C for 1 min (c). The phase-separated luminophore crystals with sharp angular edges observed in (b) can be re-dissolved and disappear upon thermally annealing the sample at a high temperature.


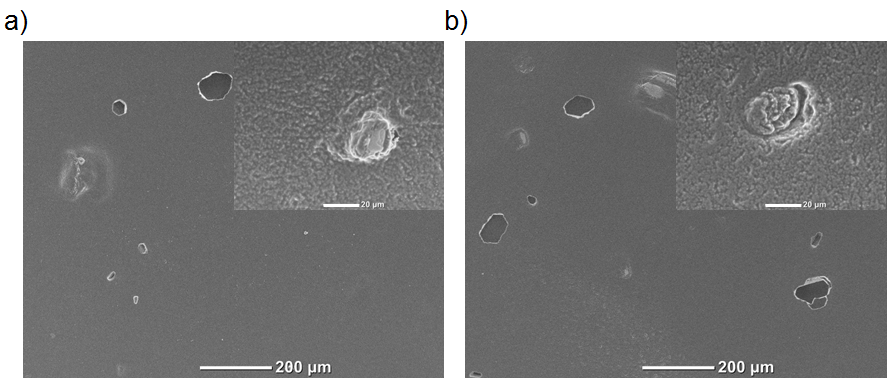


**Figure S5.** SEM images collected from P(VDF-HFP) ECL gels before (a) and after (b) the ECL measurement of applied peak to peak voltages of V_PP_ = 3.0 V. Insets show enlarged SEM images for the ECL gels. The gels look qualitatively the same and no significant changes are observed in the gel morphology.


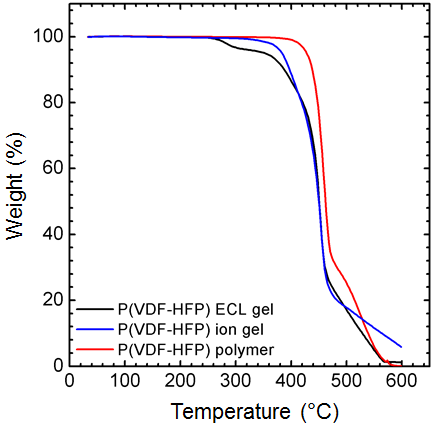


**Figure S6.** (a) TGA thermogram of the light emitting P(VDF-HFP) ECL gel, P(VDF-HFP) ion gel, and P(VDF-HFP) polymer under a nitrogen atmosphere at a heating rate of 10 °C min^−1^.


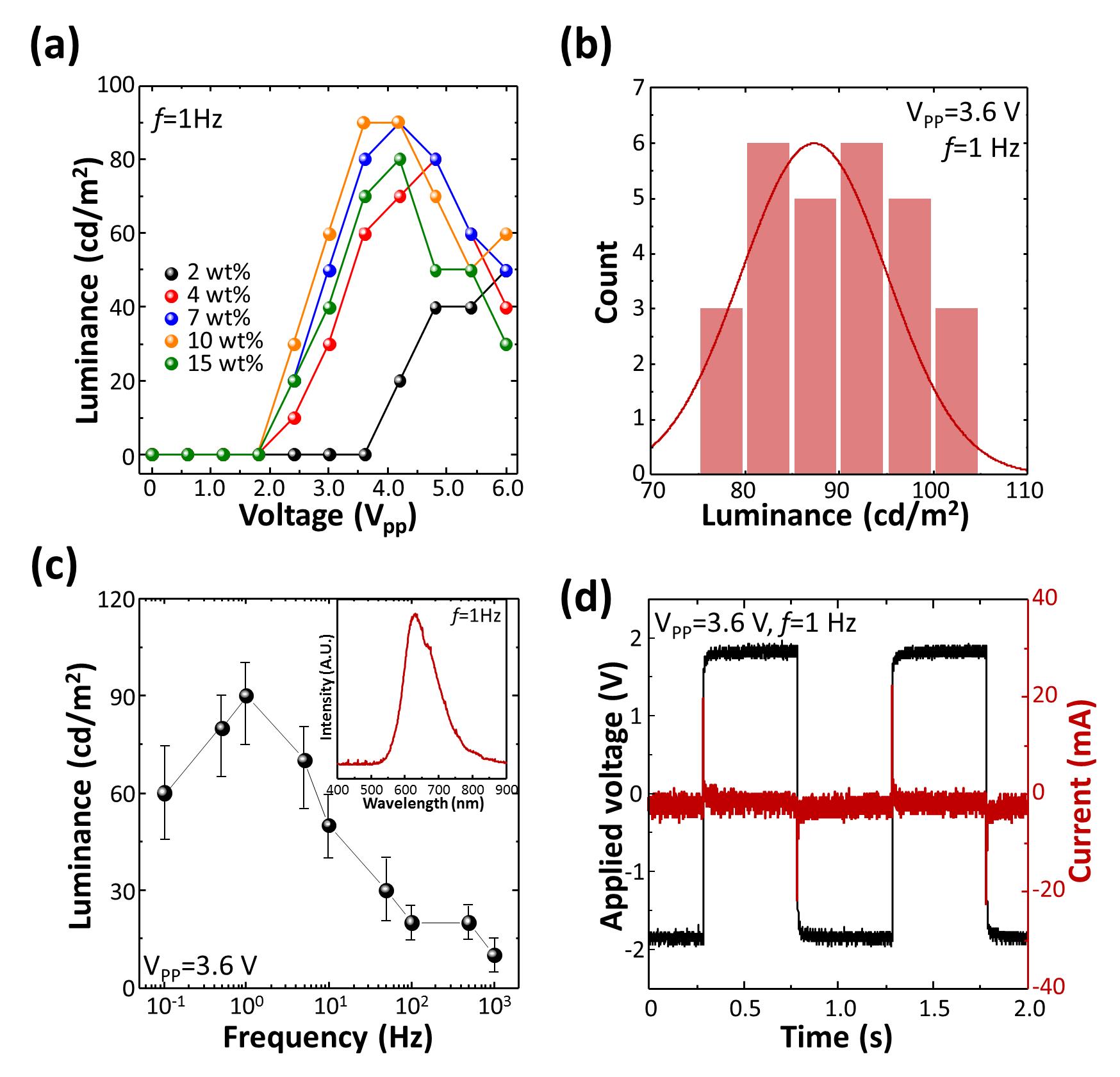


**Figure S7**. (a) Luminance-voltage characteristics of the ion gel ECLs transferred onto glass/ITO substrates with different Ru(bpy)_3_Cl_2_ concentrations. (b) Luminance statistics for 30 ECL devices attached on glass/ITO substrates. (c) Dependence of the maximum luminance on the applied voltage at AC input frequencies from 1 to 1000 Hz. (d) Current profile of the AC-driven ion gel ECL (V_pp_= 3.6 V, *f* = 1 Hz).


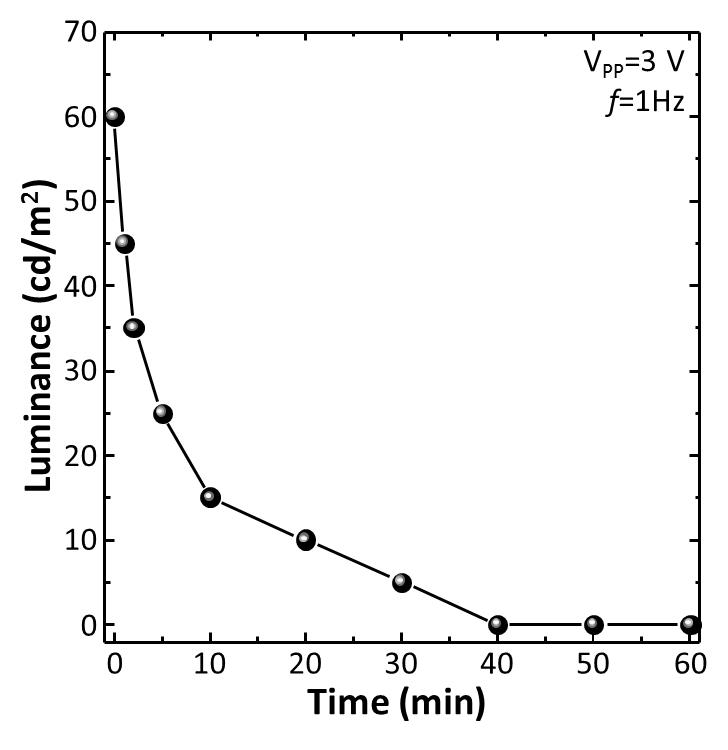


**Figure S8**. Long-term stability of ion gel based ECL devices. The measurement was performed in air ambient.


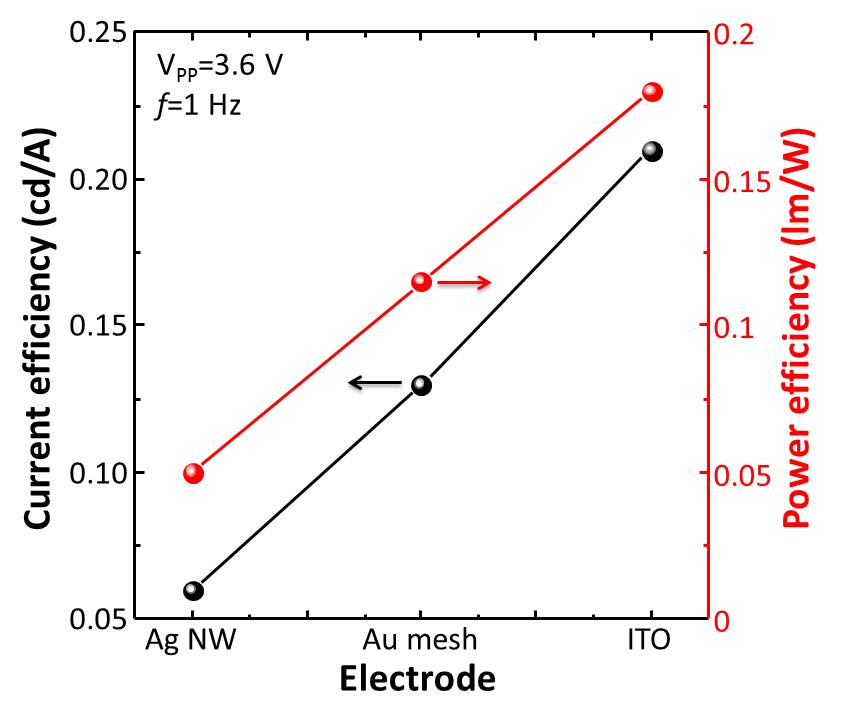


**Figure S9**. Current and power efficiencies of ECL device attached on various electrodes.


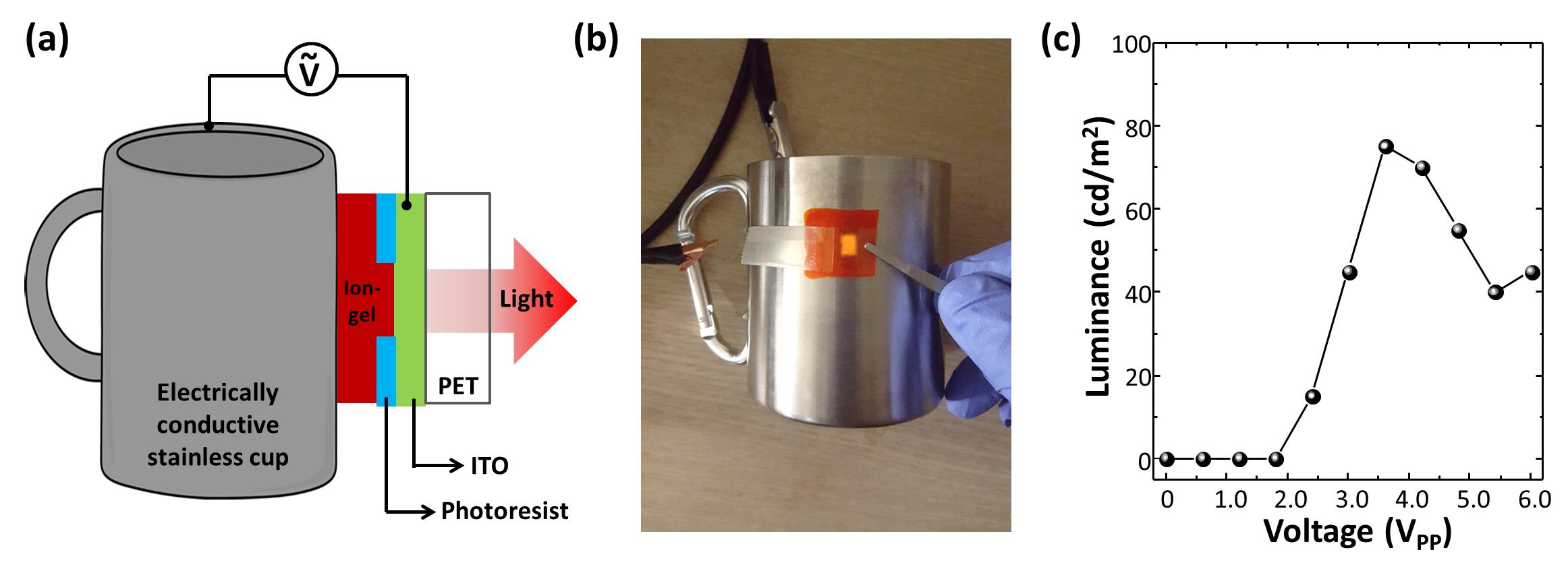


**Figure S10**. (a) Scheme and (b) photograph of a sticker-type ion gel ECL transferred onto a stainless cup. The electrically conductive stainless cup acts as a reflective electrode. An ITO-coated PET film was laminated on the top surface of the ion gel as a transparent electrode. The diagram is not to scale. (c) Luminance-voltage curve of ion gel ECL device transferred onto a stainless cup.


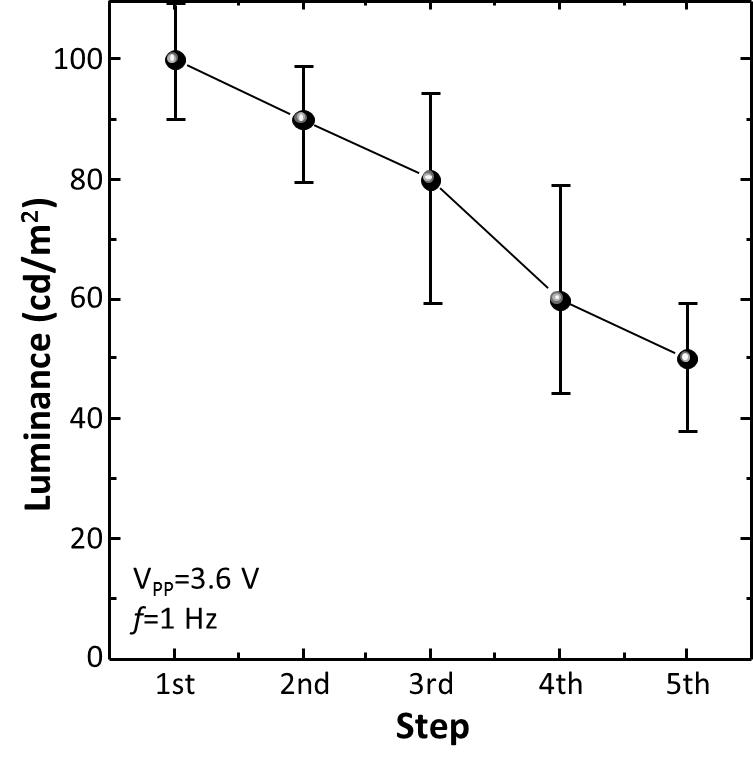


**Figure S11**. Changes in luminance for five consecutive repeatability tests.

**Supporting Information Video 1 | Sticker-type optoelectronic device.** Video demonstrating sticker-type optoelectronic devices using ion gel film. The ion gel is peeled-off from glass substrate then transfered to glass/ITO. The applied voltage (V_PP_) of AC bias and frequency were 3.6 V and 1 Hz, respectively.

**Supporting Information Video 2 | Repeatability feature of ECL device.** Video 2 shows the repeatability feature of the sticker ECL device. In the movie, ECL gels can be detached and re-adhered to the glass/ITO substrates multiple time, ca. 3 times, similar to a reusable “Post-it” note.
